# Effects of experimental bleaching agents on the mineral content of sound and demineralized enamels

**DOI:** 10.1590/1678-7757-2017-0589

**Published:** 2018-10-02

**Authors:** Vanessa Cavalli, Denise Aparecida da Rosa, Daylana Pacheco da Silva, Matheus Kury, Priscila C. S. Liporoni, Luis Eduardo S. Soares, Airton Abraão Martins

**Affiliations:** 1 Universidade Estadual de Campinas, Faculdade de Odontologia de Piracicaba, Departamento de Odontologia Restauradora, Piracicaba, São Paulo, Brasil.; 2 Consultório particular, Taubaté, São Paulo, Brasil.; 3 Universidade de Taubaté, Taubaté, São Paulo, Brasil.; 4 Universidade do Vale do Paraíba, Instituto de Pesquisa e Desenvolvimento (IP&D), Laboratório de Odontologia e Materiais Aplicados, São José dos Campos, São Paulo, Brasil.; 5 Universidade Brasil, Grupo de Espectroscopia Biomédica Vibracional, São Paulo, São Paulo; Universidade Federal do Piauí, Departamento de Física, Teresina, Piauí, Brasil.

**Keywords:** Tooth bleaching, Hydrogen peroxide, Fluorides, Tooth remineralization

## Abstract

High concentrations of hydrogen peroxide can cause adverse effects on composition and structure of teeth. However, the addition of calcium and fluoride in bleaching agents may reduce enamel demineralization.

**Objective:**

To evaluate chemical changes of sound and demineralized enamels submitted to high concentrations of hydrogen peroxide containing fluoride (F) or calcium (Ca).

**Material and Methods:**

Enamel blocks of bovine incisors with standard dimensions were obtained and half of them were submitted to pH-cycling to promote initial enamel caries lesions. Sound and demineralized enamel samples were divided into (n=10): (C) Control (no whitening treatment); (HP) 35% hydrogen peroxide; and two experimental groups: (HPF) 35% HP+0.2% F and (HPC) 35% HP+0.2% Ca. Experimental groups were submitted to two in-office bleaching sessions and agents were applied 3 times for 15 min to each session. The control group was kept in remineralizing solution at 37°C during the bleaching treatment. The surface mineral content of sound and demineralized enamels was determined through Fourier Transform Raman spectroscopy (FT-Raman), Energy dispersive Micro X-ray fluorescence spectroscopy (μ-EDXRF); and the subsurface, through cross-sectional microhardness (CSMH). In addition, polarized light microscopy (PLM) images of enamel subsurface were observed.

**Results:**

According to three-way (FT-Raman and μ-EDXRF analyses) or two-way analysis of variance (ANOVA) (CSMH) and Tukey test (α=5%), the calcium or fluoride added to high-concentrated bleaching agents increased phosphate and carbonate concentrations on sound and demineralized enamels (p<0.05). However, HPC and HPF were unable to completely reverse the subsurface mineral loss promoted by bleaching on sound and demineralized enamels. The calcium/ phosphate (Ca/P) ratio of sound enamel decreased after HP treatment (p<0.001).

**Conclusion:**

Even though experimental bleaching agents with Ca or F reduced mineral loss for both sound and demineralized enamel surfaces, these agents were unable to reverse the enamel subsurface demineralization.

## Introduction

In-office bleaching has been extensively performed clinically due to the fast whitening promoted by the higher concentrations of hydrogen peroxide (HP) in one single application. ^1^ However, low and high concentrations of HP can uphold adverse effects on enamel composition and structure. ^2^ ^,^ ^3^ Among these alterations, the decrease of enamel inorganic content and enamel cohesive strength ^4^ was observed. In addition, *in vitro* studies have demonstrated increase in surface roughness after bleaching. ^5^ ^,^ ^6^

High concentrations of HP can modify calcium/ phosphate (Ca/P) ratio ^7^ and the loss of mineral content decreases microhardness, which might increase enamel susceptibility to demineralization. ^8^ Such considerations are concerning since bleaching could interfere in the development of early caries lesions that are not properly detected. ^9^ ^-^ ^11^

The mechanism of action of the agents may be responsible for the structural enamel changes after bleaching. ^6^ It is believed that the active compound H_2_O_2_ penetrates enamel and dentin and, due to its chemical instability, it is decomposed into different active oxygen species under specific temperature, pH and light conditions. ^12^ The free radicals formed, as hydroxyl, may oxidize the conjugated chain of organic compounds, the chromophores. ^12^ The oxidation or cleavage of organic molecules that occur during bleaching possibly promotes morphological changes of the enamel. ^13^

On the other hand, fluoride has been extensively proven efficient to increase F concentration in enamel and reduce enamel demineralization. ^14^ Due to the effectiveness of re-hardening on demineralized enamel, ^14^ fluoride has been added to bleaching agents to minimize the effects of bleaching on enamel. ^7^ ^,^ ^15^ ^,^ ^16^ It is believed that high concentrations of fluoride and calcium added to the bleaching agents will control mineral loss, even if the agent has low pH, in view of the fact that the bleaching will be saturated with ions. ^15^ ^,^ ^16^

Yet, studies have observed that home-applied bleaching agents (10% carbamide peroxide), enhanced with high concentrations of fluoride (0.2%) or calcium (0.2%) applied 6 h a day for 14 days, minimized enamel mineral loss. ^7^ ^,^ ^16^ Therefore, it is possible that the addition of fluoride or calcium to high-concentrated bleaching agents (35% hydrogen peroxide) could prevent mineral loss. This possibility is particularly interesting to avoid further mineral loss in patients with early caries lesions. ^9^ ^,^ ^10^ Although the effectiveness of the addition of fluoride and calcium to bleaching agents has been evaluated, ^7^ ^,^ ^17^ the concentrations of both agents vary considerably. Previous studies attest that high concentrations of calcium (0.2%) and fluoride (0.2%) are required to prevent demineralization of bleached enamel. ^7^ ^,^ ^16^ However, these concentrations have been tested mainly in low-concentrated bleaching gels. ^7^ ^,^ ^16^ Therefore, the aim of this study was to determine mineral loss of sound and demineralized enamels submitted to experimental in-office bleaching agents containing 0.2% calcium or 0.2% fluoride. The surface and subsurface of enamel were evaluated to assess the extension of demineralization. Therefore, the tested hypotheses were that (1) the 35% hydrogen peroxide bleaching agent containing either calcium or fluoride would change the surface mineral content of sound or demineralized enamels and that (2) the addition of calcium or fluoride to 35% hydrogen peroxide would increase mineralization of enamel subsurface.

## Materials and methods

### Experimental design

Eighty dental enamel blocks obtained from bovine incisors were used in this randomized *in vitro* study and half of them (40) were submitted to pH-cycling to promote enamel demineralization. Thereafter, sound and demineralized enamel were assigned to four treatments (n = 10) as follows:

(C) Control group - without bleaching;

(HP) Commercially available 35% hydrogen peroxide agent (Whiteness HP Maxx - FGM Dental Products, Joinville, SC, Brazil) containing 35% hydrogen peroxide, thickener, pigment, neutralizing agent, glycol, distilled water and pH 6.3;

(HPF) Experimental fluoride-based bleaching agent containing 35% hydrogen peroxide with 0.2% sodium fluoride, carbopol 940, neutralizing agent, propylene glycol, distilled water and pH adjusted to 6.8;

(HPC) Experimental calcium-based bleaching agent containing 35% hydrogen peroxide, 0.2% calcium chloride, carbopol 940, neutralizing agent, propylene glycol, distilled water and pH adjusted to 7.0.

The components of experimental agents (carbopol 940, propylene glycol and distilled water) were stirred under room temperature (20°C) and homogenized. Hydrogen peroxide and 0.2% sodium fluoride (NaF) or 0.2% calcium chloride (CaCl_2_) were added to the solution. Fluoride and calcium concentrations were selected based on previous studies. ^7^ ^,^ ^15^ ^,^ ^16^ The neutralizing solution (NaOH) adjusted the pH and the experimental agent was constantly kept under refrigeration (8°C±1°C) throughout the experiment. Two bleaching sessions with an interval of seven days were performed and bleaching was applied for three times of 15 minutes each.

Mineral loss of the surface enamel was determined at baseline (T_0_) and 24 h after bleaching therapy (T_b_) through FT-Raman spectroscopy (FT-Raman) and Energy dispersive Micro X-ray fluorescence spectroscopy (μ-EDXRF). Also, after bleaching treatment, the enamel subsurface demineralization and lesion depth were respectively assessed by Knoop cross-sectional microhardness (CSMH) measurements and enamel surface was observed under polarized light microscopy (PLM).

### Specimen preparation

This study was analyzed and approved by the Ethical Research Committee of the University of Taubaté, Brazil (protocol #0033/07). Eighty sound bovine incisors were obtained and stored after extraction in deionized water with thymol granules at 5°C for no longer than 2 weeks. Teeth were cleaned and placed in deionized water for 24 h before the beginning of the experiment. Two enamel blocks (4 mm X 4 mm) were obtained from the buccal surface and enamel surfaces were flattened with wet aluminum oxide abrasive papers (#600, 800, 1,000, and 1,200) and polished with diamond pastes (6, 3, 1 and ¼µm) on a polishing machine (Arotec, Cotia, SP, Brazil). A round 7 mm^2^ area was limited on the exposed enamel with nail varnish (Colorama, São Paulo, SP, Brazil). Three microhardness indentations (hardness tester - Future Tech FM-ARS, Tokyo, Japan) were performed in each block using a Knoop diamond under a 25 g load for 5 s. Mean values were used to select enamel blocks with similar hardness. With regard to the Knoop Hardness Number (KHN) mean, eighty enamel blocks with similar microhardness values were selected (p=0.226) and half of them were submitted to pH-cycling to create early artificial caries lesions. The pH-cycling consisted of immersing specimens for 16 h in remineralizing solution [1.5 mM calcium (CaCl_2_), 0.9 mM phosphate (NaH_2_PO_4_), 0.15 mM KCl, adjusted to pH 7.0 and 3.125 mL of solution for each mm^2^ of exposed enamel] ^18^ and for 8 h in demineralizing solution (2.2 mM calcium, 2.2 mM phosphate, 1 ppm F NaF, 0.05 M acetic acid, adjusted to pH 4.5 and 6.25 mL of solution for each mm^2^ of exposed enamel) ^18^ for 8 days.

### Bleaching treatment

Bleaching agents (HP, HPF and HPC – 0.01 g) were applied on exposed enamel for 15 minutes and specimens were rinsed thoroughly. This procedure was repeated 3 times, following the instructions of the commercial product tested. At the end of the first bleaching session, specimens were immersed in remineralizing solution for seven days, and a second bleaching application was performed. Meanwhile, the control group remained immersed in remineralizing solution at 37°C. Twenty-four hours after the end of bleaching therapy, samples were submitted to FT-Raman and μ-EDXRF, which are non-destructive analyses. Seventy-two hours after the end of bleaching, samples were prepared to CSMH and PLM. Throughout the specimen preparation for tests performed, samples were kept in relative humidity.

### FT-Raman spectroscopy (FT-Raman)

The inorganic concentration (phosphate and carbonate) of enamel before and after bleaching treatments was determined through FT-Raman spectroscopy. The selection of parameters used for the FT-Raman spectrometer (RFS 100/S, Bruker Inc., Karlsruhe, Germany) and the subsequent data analyses were carried out according to previous description. ^16^

All spectra were processed by fitting the Raman vibrational modes, including phosphate (PO_4_^3-^) peaks *v_2_* (431-449 cm^-1^) and *v_4_* (582-611 cm^-1^), both in different modes, and *v_3_* (1070 cm^-1^) attributed to carbonate (CO_3_^2-^) vibration. Spectra were corrected at baseline and then normalized to the 960 cm^-1^ peak. To obtain the area of each band, the band decomposition was performed by Gaussian shapes and the mean of the peak areas were calculated by the Microcal Origin 5.0 software (Microcal Software Inc., Northampton, MA, USA).

### Micro Energy dispersive Micro X-ray fluorescence spectrometer (μ-EDXRF)

Micro energy dispersive X-ray fluorescence spectrometer (μ-EDXRF 1300, Shimadzu, Kyoto, Japan) was used to map the surface area to measure the weight of Ca/P ratios on sound and demineralized enamel surfaces before and after bleaching treatments. The selection of parameters used for the μ-EDXRF and subsequent data analyses were performed according to the method described by Paula et al. ^19^ (2010).

### Cross-sectional microhardness analysis (CSMH)

Specimens were cut longitudinally through the center of the exposed enamel area and two halves were obtained. One of the enamel segments was embedded in acrylic resin and the inner exposed surface was polished serially (#600, #800, #1000, #1200-grit sandpapers). Three rows of ten indentations each were made in the central area of the block using a microhardness tester (Future Tech FM-ARS, Tokyo, Japan) with a Knoop diamond under a 25 g load for 5 seconds at 20, 40, 60, 80, 100, 120, 140, 160, 180, 200 μm from the outer enamel surface; the distance between them was set at 100 μm. Average values at each distance from the outer surface were expressed in KHN. Values in KHN were converted into mineral volume percent: mineral volume % =4.3 KHN^1/2^+11.3. ^20^

The mineral volume percent was plotted against depth for each specimen and the integrated mineral content (Z) of the enamel lesion was calculated in comparison to the underlying sound enamel. Sound enamel values for computing the integrated mineral loss were obtained from the sound enamel under the lesion in each tooth. To calculate ΔZ values, the integrated mineral content of the lesion was subtracted from that of sound enamel (Z_sound_-Z_demineralized_) ^21^ .

### Polarized light microscopy (PLM)

The other half of the specimen was polished to obtain sections 100 μm (±10) thick for polarized light microscopy examination. Sections were arranged on a glass plate, embedded in deionized water and images were observed at a 200× magnification (DMLSP, Leica Microsystems, Heerburg, Switzerland).

### Statistical analysis

The statistical power was calculated by the SAS Power and Sample Size version 12.1. The calculated power was above 80%. The normal distribution of values was verified by Kolmogorov-Smirnov and Lilliefors tests (p>0.05) and a parametric analysis was performed. The area of phosphate (PO_4_^3-^) peaks *v_2_* (431-449 cm^-1^) and *v_4_* (582-611 cm^-1^), and carbonate (CO_3_^2-^) *v_3_* (1070 cm^-1^) of FT-Raman, Ca/P ratio of μ-EDXRF, were submitted to three way ANOVA and Tukey Test according to the factors: enamel (two levels: sound and demineralized), bleaching treatment (four levels: C, HP, HPF, HPC) and time (two levels: baseline and after bleaching). The mineral loss area (ΔZ) obtained in CSMH evaluation was submitted to two way ANOVA and Tukey Test, according to the factors: enamel (two levels: sound and demineralized), bleaching treatment (four levels: C, HP, HPF, HPC). A significance level of 5% was set for all analyses and the SAS 9.0 software (SAS Institute, Cary, NC, USA) was used.

## Results

### FT-Raman spectroscopy

**Figure 1:**
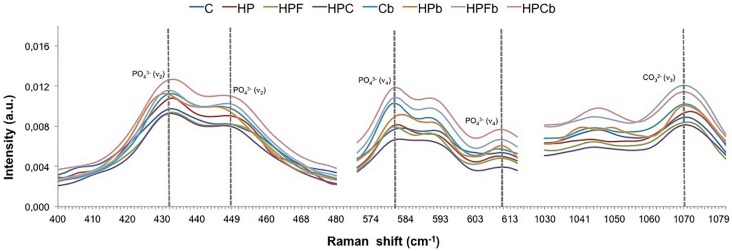
FT-Raman average spectra of mineral components in the 400-1.080cm^-1^ region showing phosphate peaks ( *v* 2, 431-449 cm^-1^ and *v4,* 582-611 cm^-1^) and carbonate peak *v* 3 (1070 cm^-1^) of sound enamel before (C, HP, HPF and HPC) and after (Cb, HPb, HPFb and HPCb) bleaching. It can be noted that after bleaching, groups C, HPF and HPC increased phosphate ( *v* 2 and *v* 4) and carbonate ( *v* 3) contents of enamel, while HP-treated enamel remained with similar inorganic composition

Sound enamel: At baseline, the phosphate concentration in 431-449 cm^-1^ ( *v_2_* ) and 582-611 cm^-1^ ( *v_4_* ) stretching modes for sound enamel was similar for all groups evaluated ( Table 1 , p=0.227 and Table 2 , p=0.325). After bleaching, phosphate peaks ( *v_2_* and *v_4_* ) of HP-treated group were similar to those of baseline ( Table 1 , p=0.182 and Table 2 , p=0.197), but lower than groups C, HPF and HPC (p<0.001). In addition, the phosphate concentration ( *v_2_* and *v_4_* ) of groups C, HPF and HPC increased after bleaching (p<0.0001, Table 1: *v_2_* , Table 2: *v_4_,* Figure 1 ).

**Table 1:** Mean values and standard deviations of relative areas of phosphate (PO_4_^3-^) peak *v2* (431-449 cm^-1^) (in arbitrary units) of FT-Raman spectra of sound and demineralized enamel at baseline (T_0_) and after bleaching (T_b_)

Treatments	Sound enamel				Demineralized enamel			
	^T^_0_		^T^b		^T^_0_		^T^b	
C	3.75	(0.12)^Aa^	5.89	(0.92)^Ab^	1.55	(0.47)^Aa^ *	6.49	(0.76)^Ab^
HP	3.69	(0.18)^Aa^	3.57	(0.33)^Ba^	1.87	(0.92)^Aa^ *	4.79	(0.41)^Bb^ *
HPF	3.57	(0.17)^Aa^	6.13	(0.59)^Ab^	1.75	(0.75)^Aa^ *	6.36	(0.51)^Ab^
HPC	3.46	(0.22)^Aa^	6.89	(0.97)^Ab^	1.98	(0.67)^Aa^ *	6.57	(0.12)^Ab^

Mean values followed by distinct letters differ statistically at 5%, according to three-way ANOVA and Tukey test (p<0.05). Uppercase letters compare treatments (in columns) and lowercase letters compare time (in row).

Asterisks (*) compare each enamel condition within time

C: control HP: 35% hydrogen peroxide HPF: fluoride-based bleaching agent containing 35% hydrogen peroxide HPC: calcium-based bleaching agent containing 35% hydrogen peroxide

**Table 2:** Mean values and standard deviations of relative areas of phosphate (PO_4_^3-^) peak *v4* (582-611 cm^-1^) (in arbitrary units) of FT-Raman spectra of sound and demineralized enamel at baseline (T_0_) and after bleaching (T_b_)

Treatments	Sound enamel				Demineralized enamel			
	^T^_0_		^T^b		^T^_0_		^T^b	
C	3.69	(0.16)^Aa^	5.77	(0.32)^Ab^	1.38	(0.65)^Aa^ *	4.32	(0.26)^Ab^ *
HP	3.12	(0.29) ^Aa^	2.89	(0.55)^Ba^	1.73	(0.81)^Aa^ *	4.55	(0.31)^Ab^ *
HPF	3.31	(0.09) ^Aa^	5.99	(0.39)^Ab^	1.89	(0.35)^Aa^ *	5.79	(0.35)^Bb^
HPC	3.17	(0.11) ^Aa^	6.72	(0.87)^Ab^	1.77	(0.87)^Aa^ *	5.89	(0.58)^Bb^

Mean values followed by distinct letters differ statistically at 5%, according to three-way ANOVA and Tukey test (p<0.05). Uppercase letters compare treatments (in columns) and lowercase letters compare time (in row).

Asterisks (*) compare each enamel condition within time

C: control HP: 35% hydrogen peroxide HPF: fluoride-based bleaching agent containing 35% hydrogen peroxide HPC: calcium-based bleaching agent containing 35% hydrogen peroxide

The carbonate (CO_3_^2-^, *v*_3_, 1070 cm^-1^) concentration of sound enamel was similar among groups (p<0.05), but increased after treatment with C, HPF and HPC ( Table 3 , Figure 1 ). On the other hand, HP-treated group had the lowest CO_3_^2-^ concentration among groups, after treatments (p<0.001).

**Table 3:** Mean values and standard deviations of relative areas of carbonate (CO_3_^2-^) peak *V3* (1070 cm^-1^) (in arbitrary units) of FT-Raman spectra of sound and demineralized enamel at baseline (T_0_) and after bleaching (T_b_)

Treatments	Sound enamel				Demineralized enamel			
	T_0_		T_b_		T_0_		T_b_	
C	3.66	(0.16)^Aa^	5.51	(0.22)^Ab^	2.19	(0.32)^Aa^ *	5.39	(0.56)^Ab^ *
HP	3.56	(0.29) ^Aa^	3.13	(0.24)^Ba^	1.95	(0.44)^Aa^ *	4.11	(0.31)^Bb^ *
HPF	3.57	(0.15) ^Aa^	5.23	(0.47)^Ab^	1.97	(0.28)^Aa^ *	5.42	(0.63)^Ab^
HPC	3.49	(0.21) ^Aa^	6.55	(0.89)^Ab^	2.07	(0.77)^Aa^ *	5.38	(0.88)^Ab^

Mean values followed by distinct letters differ statistically at 5%, according to three-way ANOVA and Tukey test (p<0.05). Uppercase letters compare treatments (in columns) and lowercase letters compare time (in row).

Asterisks (*) compare each enamel condition within time

C: control HP: 35% hydrogen peroxide HPF: fluoride-based bleaching agent containing 35% hydrogen peroxide HPC: calcium-based bleaching agent containing 35% hydrogen peroxide

**Figure 2:**
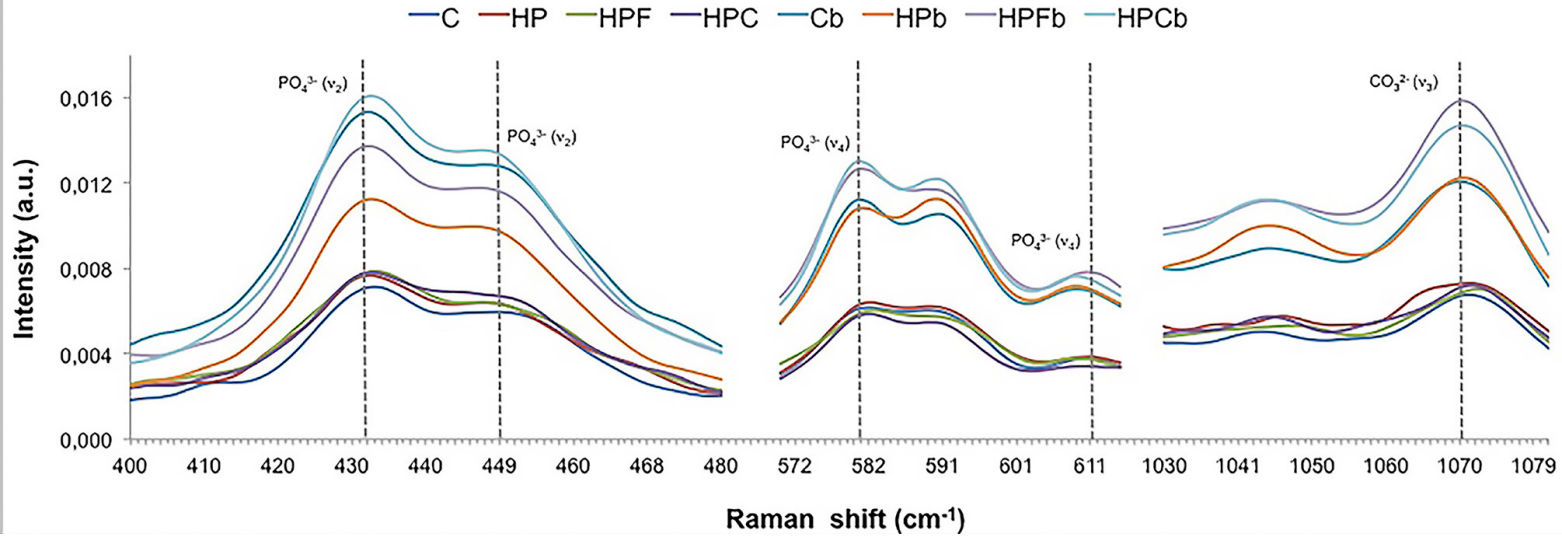
FT-Raman average spectra of mineral components in the 400-1.080cm^-1^ region showing phosphate peaks *v* 2 (431—449cm^-1^ and *v4,* 582-611cm^-1^) and carbonate peak *v* 3 (1070 cm-1) of demineralized enamel before (C, HP, HPF and HPC) and after (Cb, HPb, HPFb and HPCb) bleaching. It can be noted that after bleaching, all groups tested increased phosphate ( *v* 2 and *v4)* and carbonate ( *v* 3) inorganic contents of enamel

Demineralized enamel: At baseline, phosphate concentrations in stretching modes *v_2_* (431-449 cm^-1^) and *v_4_* (582-611 cm^-1^) of demineralized enamel were similar for all groups ( Table 1 , p=0.113 and Table 2 , p=0.2678). After bleaching, phosphate concentration of both phosphate peaks increased for all groups tested (p<0.001); however, the HP-treated group showed the lowest phosphate *v_2_* concentrations among groups after treatments (p<0.001, Table 1 , Figure 2 ). Phosphate ν_4_ concentrations of HPF and HPC groups were similar (p=0.0675) but higher than the ones of C and HP groups, after treatments (p<0.001, Table 2 , Figure 2 ).

At baseline, the carbonate (CO_3_^2-^) concentration of demineralized enamel was similar among groups (p<0.0001), but increased after treatment for all groups (C, HP, HPF, HPC ( Table 3 , Figure 2 ). However, after treatments, HP-treated group showed the lowest CO_3_^2-^ concentration among groups (p<0.001).

Sound versus demineralized enamel: Demineralized enamel had lower phosphate concentration at *v_2_* and *v_4_* vibrational modes as well as lower carbonate concentrations at *v_3_* vibrational modes than sound enamel at baseline (p<0.001), regardless of treatments. Demineralized enamel submitted to HP bleaching had higher phosphate concentration at the *v_2_* mode, than that of sound enamel (p<0.05). Demineralized enamel treated with HP or C (T_b_) had lower phosphate concentration at *v_4_,* and lower carbonate concentrations at *v*_3_ vibrational modes compared to sound enamel. Demineralized enamel submitted to HPF and HPC had similar phosphate (both at *v_2_* and *v_4_* vibrational modes) and carbonate concentrations *(v_3_)* than those of sound enamel (p>0.05).

### μ-EDXRF

Sound enamel: At baseline, sound enamel showed similar Ca/P ratios for all groups tested (p=0.177, Table 4 ). After bleaching, the Ca/P ratio of HP-treated group decreased (p<0.0001), HPF- and HPC-treated groups increased (p<0.001) and the mineral content of the C group was similar to baseline (p=0.069). HPF- and HPC-treated groups had the highest Ca/P ratio among groups at T_b_ as the HP group had the lowest (p<0.001).

**Table 4:** Mean values and standard deviations of sound and demineralized enamel Ca/P weight ratios at baseline (T_0_) and after bleaching (T_b_) (weight percentage, wt %), according to μ-EDXRFresults

Groups	Sound enamel				Demineralized enamel			
	T_0_		T_b_		T_0_		T_b_	
C	1.97	(0.11)^Aa^	1.98	(0.07)^Ba^	1.28	(0.04)^Aa^ *	1.96	(0.04)^Ab^
HP	1.99	(0.07)^Aa^	1.67	(0.05)^Cb^	1.32	(0.05)^Aa^ *	1.68	(0.09)^Ba^
HPF	1.92	(0.02)^Aa^	2.29	(0.05)^Ab^	1.22	(0.06)^Aa^ *	2.02	(0.08)^Ab^ *
HPC	1.94	(0.06)^Aa^	2.32	(0.07)^Ab^	1.20	(0.05)^Aa^ *	1.99	(0.04)^Ab^ *

Mean values followed by distinct letters differ statistically at 5%, according to three-way ANOVA and Tukey test (p<0.05). Uppercase letters compare treatments (in columns) and lowercase letters compare time (in row).

Asterisks (*) compare each enamel condition within time

C: control HP: 35% hydrogen peroxide HPF: fluoride-based bleaching agent containing 35% hydrogen peroxide HPC: calcium-based bleaching agent containing 35% hydrogen peroxide

Demineralized enamel: At baseline, all groups had similar Ca/P ratios (p = 0.188, Table 4 ). After bleaching, the Ca/P ratio of HP group was similar to T_0_ but significantly increased for C-, HPF- and HPC- treated groups (p<0.0001). After bleaching, groups C, HPF and HPC had higher Ca/P ratios than HP group (p<0.001).

Sound versus demineralized enamel: At baseline, the demineralized enamel had lower Ca/P ratio than that of sound enamel (p<0.001). After treatments, demineralized enamel submitted to HPF and HPC treatments showed lower Ca/P ratios than that of sound enamel (p<0.001).

### Cross-sectional microhardness analysis (CSMH)

Sound enamel: The mineral loss area (ΔZ) of sound C group was significantly lower than HP, HPF and HPC (p<0.001). No differences were observed among HP, HPF, HPC groups (p=0.135, Table 5 ).

**Table 5:** Mean values and standard deviations of enamel mineral loss area (ΔZ) of sound and demineralized enamel

Groups	ΔZ				
	Sound enamel			Demineralized enamel	
Control	199.3	(57.8)^Aa^		578.4	(74.7)^Ab^
HP	565.3	(87.2)^Ba^		962.6	(121.8)^Cb^
HPF	494.3	(51.9)^Ba^		842.4	(91.7)^Bb^
HPC	509.4	(45.7)^Ba^		868.8	(69.6)^Bb^

Mean values followed by distinct letters differ statistically at 5%, according to three-way ANOVA and Tukey test (p<0.05). Uppercase letters compare treatments (in columns) and lowercase letters compare time (in row).

Asterisks (*) compare each enamel condition within time

C: control HP: 35% hydrogen peroxide HPF: fluoride-based bleaching agent containing 35% hydrogen peroxide HPC: calcium-based bleaching agent containing 35% hydrogen peroxide

Demineralized enamel: After HP treatment, the demineralized enamel presented greater ΔZ than the other groups (p<0.001, Table 5 ). Groups HPF and HPC showed intermediate result, whereas group C had the lowest ΔZ.

Sound versus demineralized enamel: Demineralized enamel had greater ΔZ than sound enamel, regardless of the treatment performed (p<0.001).

### Polarized light microscopy (PLM)

**Figure 3:**
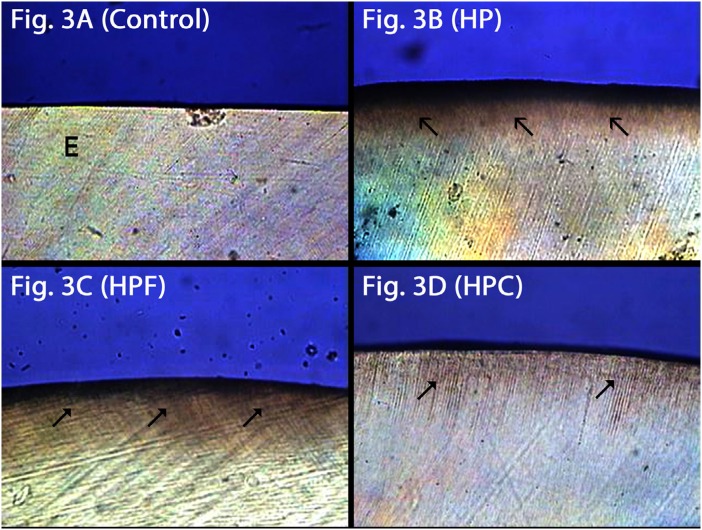
Sound enamel through polarized light microscopy. 3A: Control group with sound surface; 3B: HP group with slightly demineralized area on the surface; 3C: (HPF) minor subsurface demineralization similar to Figure 3D (HPC). (E: enamel; black arrows: demineralized area)

Sound enamel: C group ( Figure 3A ) showed surface with no signs of demineralization, while HP had a slight demineralized area at the surface ( Figure 3B ). Groups HPF ( Figure 3C ) and HPC ( Figure 3D ) showed subsurface demineralization, but the mineral loss pattern denoted an intermediary phase compared to HP ( Figure 3B ).

**Figure 4:**
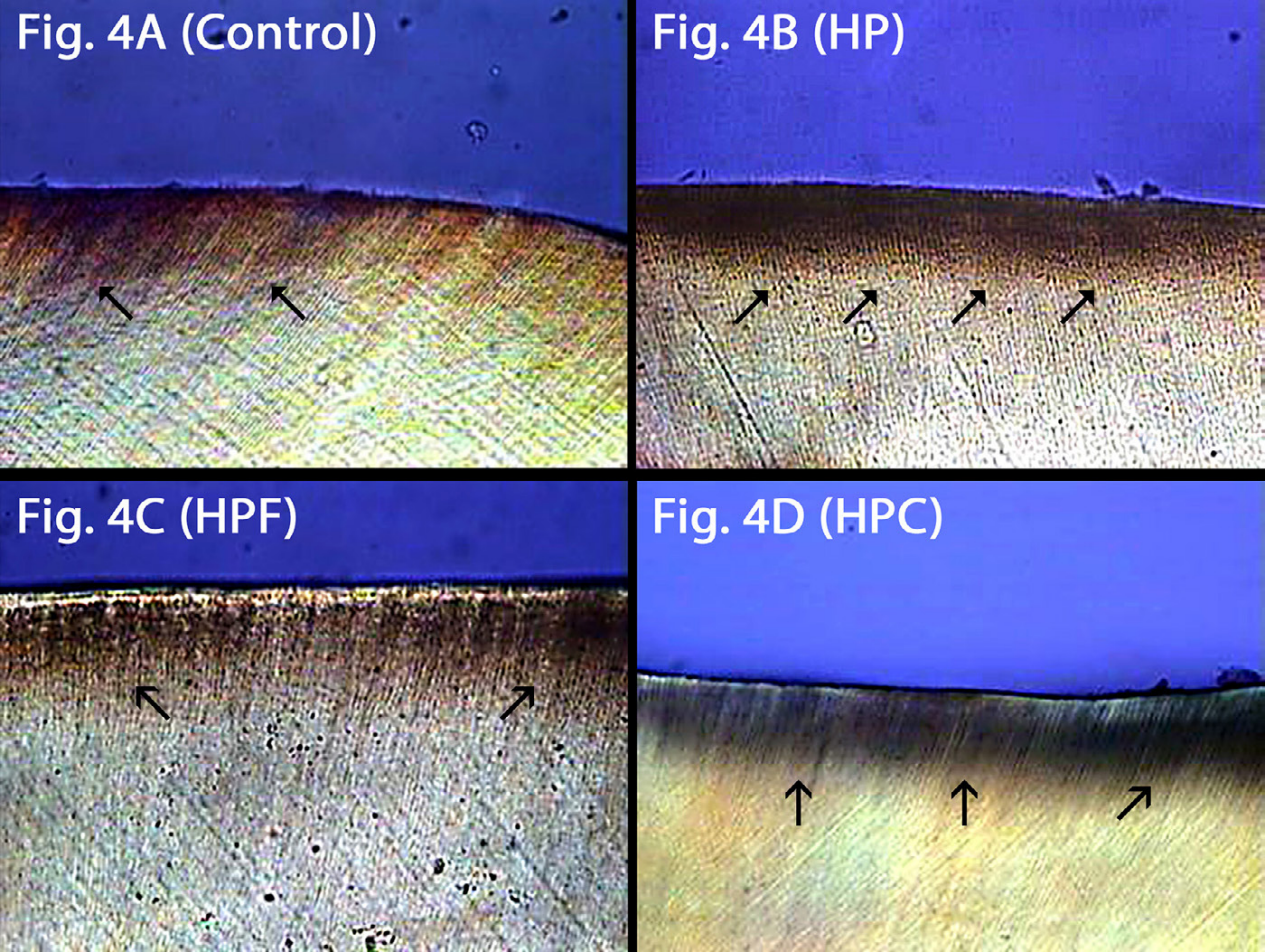
Demineralized enamel through polarized light microscopy. 4A: Control group with signs of surface demineralization due to the previous pH-cycling; 4B (HP): Extensive demineralized enamel subsurface area promoted by pH-cycling and bleaching treatment; 4C (HPF): subsurface demineralization similar to Figure 4D (HPC). (black arrows: demineralized area)

Demineralized enamel: Demineralization on enamel surface can be noted for all groups due to the pH-cycling before the bleaching treatment ( Figures 4A , 4B , 4C and 4D ). However, HP group ( Figure 4B ) showed a slightly more prominent demineralization that the other groups ( Figures, 4A , 4C , 4D ), with intense and continuous demineralized area on the enamel surface. It can be observed that the control group had discontinuation of such demineralized areas, and HPF and HPC groups showed that the top enamel surface was not as demineralized as the enamel subsurface.

## Discussion

This investigation analyzed the behavior of sound and demineralized enamels (incipient-caries like lesions) submitted to bleaching with experimental agents containing calcium and fluoride. The purpose was to verify the efficacy of experimental gels in inhibiting or controlling mineral loss of enamel in both conditions.

The FT-Raman spectroscopy detects phosphate and carbonate associated with hydroxyapatite, according to the vibration modes of each component: phosphate (PO_4_^3^) peaks *v_2_* (431-449 cm^-1^) and *v_4_* (582-611 cm^-1^), and carbonate (CO_3_^2-^) *v_3_* (1070 cm^-1^). On the other hand, μ-EDXRF analysis indicates the Ca/P ratio in both sound and demineralized enamels before and after bleaching. At baseline, sound enamel groups had similar phosphate, carbonate and Ca/P concentrations, and the same was observed for demineralized groups. However, 35% hydrogen peroxide with F (HPF) or Ca (HPC) increased phosphate ( *v*_2_ and *v*_4_) and carbonate *(v_3_)* concentrations after bleaching and the mineral content was higher than that of HP-treated group ( Tables 1 , 2 and 3 ). Nevertheless, μ-EDXRF analysis showed that HP bleaching reduced the Ca/P ratio on sound enamel ( Table 4 ), which indicates that HP alone could compromise enamel inorganic concentration. According to this scenario, F and Ca ions possibly upturned or controlled mineral loss promoted by HP. Although this is an *in vitro* evaluation and we cannot extrapolate to a clinical condition, these results signalize that HP could promote demineralization, as well as F and Ca could remineralize enamel surface after bleaching.

A previous investigation reported that the addition of 0.2% F or 0.2% Ca to home-applied bleaching agents controlled enamel inorganic loss on intact enamel surfaces compared to conventional 10% carbamide peroxide gels. ^16^ According to these findings, the effectiveness of enhanced bleaching agents is attributed to the high concentrations of F and Ca in experimental bleaching agents as an ionic modulation occurring between the undersaturated enamel surface and the saturated bleaching gel. ^16^

The decreased Ca/P ratio of HP-treated groups detected only by μ-EDXRF could be explained by the mechanism of action of the hydrogen peroxide. According to Park, et al. ^22^ (2004), enamel submitted to 30% hydrogen peroxide decreases in both organic (2.800-3.000 cm^-1^) and inorganic (429-1.069 cm^-1^) vibrational modes. The low molecular weight of hydrogen peroxide enables its diffusion into enamel and interprismatic spaces, where it decomposes, releasing free radicals that can react with the organic components. ^22^ ^,^ ^23^ Since the organic content (as proteins, lipids and teeth-staining substances), is distributed along inter-zones of inorganic structures, the decomposition of these substances results in enamel surface irregularities. ^22^ In the decomposition process, HP free radicals can also interact with inorganic elements, by gradual dissolution of enamel surface by removal of mineral elements, ^22^ affecting enamel integrity and promoting carbonate loss, ^23^ removal of enamel prism core ^6^ , which increases porosity ^24^ and decreases mineral content of the enamel. ^25^

According to Coceska, et al. ^26^ (2016), the enamel bleaching with 35% HP without light activation promoted loss of sodium (Na) and magnesium (Mg), whereas, the application of laser light increased the loss of calcium (Ca) and phosphate (P) in the enamel surface. In fact, these authors confirm that high-concentrated whitening agents damage enamel surface. On the other hand, they also showed that enamel changes are reversible and can be repaired by the application of remineralizing toothpastes. In this context, fluoride remains as the most important agent to promote mineral balance, reducing demineralization and activating remineralization. ^27^ This is attributed to the formation of fluorohydroxyapatite in dental tissues ^27^ , which is less soluble in acidic solutions than hydroxyapatite. ^28^ Although the pH of experimental bleaching agents tested was close to 7.0, if the enamel was submitted to bleaching agents with low pH, it is likely that fluorapatite formed on enamel surface would remain.

FT-Raman detected increases in phosphate *(ν_2_* and *ν_4_)* and carbonate *(ν_3_)* concentrations of control group, regardless of the enamel condition (sound or demineralized enamels). Since no treatment was performed in this group, except for the storage in the remineralizing solution, which was used to mimic the clinical condition, it seems feasible to attest that the remineralizing effect of the solution promoted ionic deposition on enamel surface. ^29^ Furthermore, it must be acknowledged that the remineralizing solution might have had a synergistic effect as it increased phosphate and carbonate concentrations for both sound and demineralized enamels treated with HPF and HPC. Although μ-EDXRF detected no increase in the Ca/P ratio of control group, no changes were observed at the final measurement (T_b_). However, the Ca/P ratio after bleaching of HPF and HPC were greater than that observed for the control group, and this could support the efficacy of adding F and C to the bleaching agents.

The μ-EDXRF captured a reduction in the Ca/P ratio of sound enamel treated with HP after two bleaching sessions. Contrary to these findings, Furlan, et al. ^17^ (2017) observed that enamel microhardness decreased only after the third bleaching session with high-concentrated bleaching agents, regardless of the bleaching agent used (with or without fluoride or calcium). Moreover, they observed that bleaching agents containing sodium fluoride or calcium gluconate had higher microhardness values than 35% hydrogen peroxide alone ^17^ . According to these authors, calcium gluconate in high-concentrated agents seemed to have a positive effect on enamel, however, this effect was not observed for the low-concentrated agent. They explain that the effects of calcium gluconate are still on debate as this agent is incompatible with strong oxidizing agents. The authors report that free radicals released during bleaching (free oxygen and perhydroxyl) could be inhibited by the calcium gluconate. ^17^ In the current research, instead of calcium gluconate, calcium chloride was used. Although HPC-treatment increased phosphate and carbonate concentrations, as well as Ca/P ratio, the interaction of calcium chloride and free radicals was not chemically evaluated.

The phosphate *(ν_2_* and *ν_4_)* , carbonate *(ν_3_),* and Ca/P concentrations of demineralized enamel increased after treatments, except for the Ca/P concentration of the HP group, which remained with similar mineral concentration after bleaching ( Table 4 ). The response of demineralized enamel toward treatments (HPF and HPC) relies on the action of F and Ca deposition on the undersaturated enamel surface and the action of the remineralizing solution, since C group also had increase in the inorganic concentration. It must be noted that F and Ca uptake of demineralized enamel was higher than sound enamel, because in the first condition the enamel was undersaturated regarding the ionic concentration, which balanced the content of the parts involved (substrate, remineralizing solution and/ or bleaching agent). ^7^ Therefore, the first hypothesis can be accepted, as the 35% hydrogen peroxide agent containing calcium or fluoride increased the mineral content of sound and demineralized enamels.

CSMH and PLM provided evidence of enamel subsurface demineralization. In this specific region, demineralization could be noted for both sound and demineralized enamels. However, the mean for the mineral loss of sound enamel of the control group was lower compared to the other treatments. As expected, samples submitted to previous demineralization showed subsurface demineralization, but control group had lower demineralization area, followed by samples submitted to HPF and HPC and finally, HP bleaching agent, which presented the greatest mineral loss. A similar finding ^16^ showed that enamel submitted to 10% carbamide peroxide (CP) had enamel subsurface demineralization, which was significantly greater and deeper than that observed for the placebo group (without CP), followed by 10% CP with F or Ca. Although F or Ca were unable to completely reverse the inner demineralization, the agents could control it in that report, ^6^ different from the present study. Therefore, the second hypothesis was rejected, as the addition of Ca or F to the 35% HP bleaching agent was unable to increase mineral concentration of enamel subsurface.

Based on the exposed, the addition of F and Ca to the 35% HP agent could keep and increase the mineral content of enamel surface for both sound and demineralized substrates, but could not reverse subsurface enamel demineralization. This outcome confirms that the F and/or Ca associated with bleaching decrease enamel mineral loss of the enamel surface. ^16^ ^,^ ^30^ In addition, previous findings observed that F could not only control the mineral loss, but also did not compromise the whitening efficacy. ^31^ ^,^ ^32^

One might say that enamel mineral loss observed after bleaching may not be threatening to dental hard tissues, since it has been shown that enamel exposed to acidic beverages for some minutes promote similar alterations on enamel surface. ^33^ However, if a patient with early caries lesions undergoes bleaching therapy, its consequences could be intensified. For those patients, the addition of F or Ca could be an interesting therapy choice. Besides, it should be kept in mind that only two bleaching sessions were performed and, possibly, more bleaching sessions would increase the severity of enamel mineral loss.

## Conclusion

The addition of Ca and F to experimental bleaching agents could control the decrease of phosphate and carbonates for both sound and enamel surfaces. On the other hand, experimental bleaching agents were unable to reverse the subsurface demineralization of sound and demineralized enamels.
